# Immediate large-scale cortical network changes following acupuncture in patients with disorders of consciousness: a resting-state fNIRS study

**DOI:** 10.3389/fnins.2026.1917107

**Published:** 2026-09-18

**Authors:** Yifei Wang, Chenyang Gao, Ying Han, Jinling Zhang, Junying Wang, Shuai Han, Huan Ouyang, Lanyu Jia, Weihang Zhai, Jianghong He, Peijing Rong

**Affiliations:** 1 Institute of Acupuncture and Moxibustion, China Academy of Chinese Medical Sciences, Beijing, China; 2 Institute of Biomedical Engineering, Chinese Academy of Medical Sciences and Peking Union Medical College, Tianjin, China; 3 Department of Neurosurgery, Beijing Tiantan Hospital, Capital Medical University, Beijing, China; 4 Institute of Clinical Basic Medicine of Chinese Medicine, China Academy of Chinese Medical Sciences, Beijing, China; 5 Tianjin Academy of Traditional Chinese Medicine Affiliated Hospital, Tianjin, China

**Keywords:** acupuncture, brain networks, disorders of consciousness, fNIRS, functional connectivity, graph theory

## Abstract

**Introduction:**

Disorders of consciousness (DOC) involve severe disruption of large-scale brain networks. This study investigated immediate cortical network changes following acupuncture in patients with DOC using functional near-infrared spectroscopy (fNIRS).

**Methods:**

Thirty patients with DOC underwent resting-state fNIRS recordings immediately before and after a single acupuncture session at Shuigou (GV26), Yintang (EX-HN3), and Baihui (GV20). Oxygenated hemoglobin signals were analyzed using functional connectivity and graph-theoretical metrics.

**Results:**

Mean positive functional connectivity, global efficiency, and clustering coefficient were significantly higher after acupuncture. Posterior, within-left-hemisphere, and fronto-posterior connectivity also increased significantly. Higher nodal strength was observed in the left Broca area, bilateral primary somatosensory cortex, and right premotor cortex. Individual edge-wise changes were not significant after multiple-comparison correction.

**Discussion:**

A single acupuncture session was followed by immediate differences in large-scale cortical network organization, characterized by higher global integration and stronger sensorimotor-language and fronto-posterior connectivity. These findings reflect an acute cortical response temporally associated with acupuncture; controlled longitudinal studies are needed to determine acupuncture specificity and clinical relevance.

## Introduction

Disorders of consciousness (DOC) represent a group of severe neurological conditions in which arousal and awareness are markedly impaired after acquired brain injury. These conditions include coma, vegetative state/unresponsive wakefulness syndrome (VS/UWS), and minimally conscious state (MCS). In clinical practice, the assessment of residual consciousness remains difficult because behavioral responses are often weak, inconsistent, or absent. Beyond bedside examination, converging evidence from neuroimaging and neurophysiology indicates that consciousness depends on coordinated activity within distributed brain networks, including thalamo-cortical, frontoparietal, sensorimotor, and associative systems (Edlow et al., 2021; Kondziella et al., 2020; Shine et al., 2023). This network-based view has increased the need for objective neuroimaging biomarkers that can capture intervention-related changes in brain function and support the evaluation of consciousness rehabilitation in patients with DOC (Wang et al., 2023).

Acupuncture has been used as an adjunctive intervention in neurological rehabilitation and has also been applied clinically in patients with prolonged DOC (Wan et al., 2024; Liu et al., 2022; Xin et al., 2022). Wake-promoting acupuncture protocols commonly include craniofacial and midline Governor Vessel acupoints, such as Shuigou (GV26), Yintang (EX-HN3), and Baihui (GV20). These points are traditionally used in approaches intended to “awaken the mind” and may provide somatosensory input from the face and scalp. From a neurophysiological perspective, such peripheral stimulation may influence arousal-related systems through ascending somatosensory pathways, brainstem-autonomic interactions, and large-scale cortical network modulation. However, immediate cortical network changes following acupuncture in DOC remain insufficiently characterized.

Functional near-infrared spectroscopy (fNIRS) is particularly suitable for studying brain responses in patients with DOC. Compared with functional magnetic resonance imaging and positron emission tomography, fNIRS is portable, non-invasive, bedside-compatible, and more feasible for repeated assessment in clinically fragile patients who cannot tolerate transportation or remain stable in conventional scanning environments (Pinti et al., 2020; Wang et al., 2025). Compared with electroencephalography, fNIRS provides complementary hemodynamic information by measuring changes in oxygenated hemoglobin related to cortical blood flow regulation. These practical and physiological advantages make fNIRS well suited for capturing short-term cortical responses before and after an intervention. Resting-state fNIRS further allows the assessment of spontaneous low-frequency hemodynamic fluctuations, interregional functional connectivity, and graph-theoretical properties of cortical networks (He et al., 2024; Pinti et al., 2019). Previous fNIRS studies have further demonstrated its utility for detecting residual consciousness or cognition and for characterizing intervention-related hemodynamic responses in patients with DOC (Li et al., 2021; Si et al., 2023; Kazazian et al., 2024; Liu et al., 2025; Peng et al., 2025).

A major difficulty in interpreting brain changes observed around acupuncture in DOC is the potential influence of spontaneous fluctuation, natural recovery, medication, routine rehabilitation, and general clinical care. Changes observed after days or weeks of treatment are clinically meaningful but may be affected by multiple concurrent factors. An immediate within-subject pre−/post-acupuncture fNIRS design can reduce interindividual variability and limit the influence of longer-term clinical confounders by recording brain activity within a short time window around the acupuncture procedure, but it cannot by itself distinguish acupuncture-specific effects from spontaneous short-term fluctuations or nonspecific procedural effects. In the present study, we analyzed resting-state fNIRS data obtained immediately before and after acupuncture at GV26, EX-HN3, and GV20 in 30 patients with DOC. We aimed to characterize acute pre−/post-acupuncture differences in cortical functional connectivity and graph-theoretical network organization and to explore whether these differences show regional patterns across anterior–posterior, hemispheric, and sensorimotor–language subnetworks.

## Materials and methods

### Study design and participants

This exploratory within-subject fNIRS study was conducted to characterize acute cortical network changes surrounding a single acupuncture session in patients with prolonged DOC. The study protocol was registered in the International Traditional Medicine Clinical Trial Registry (ITMCTR2025002104) and approved by the Ethics Committee of the Institute of Acupuncture and Moxibustion, China Academy of Chinese Medical Sciences (Approval No. S2024-01-31-13). All procedures were carried out in accordance with the Declaration of Helsinki. Because the enrolled patients had impaired consciousness and were unable to provide consent themselves, written informed consent was obtained from their legally authorized representatives before study participation.

Participants were recruited from the Neurosurgical Disorders of Consciousness Unit at Beijing Tiantan Hospital, Capital Medical University. Participants were enrolled between March 2025 and April 2026.

The inclusion criteria were: (1) a diagnosis of vegetative state/unresponsive wakefulness syndrome (VS/UWS) or minimally conscious state (MCS), established by experienced clinicians on the basis of repeated Coma Recovery Scale–Revised (CRS-R) assessments (Giacino et al., 2004, 2014); (2) acquired brain injury caused by traumatic brain injury, cerebral hemorrhage, or anoxic–ischemic encephalopathy; (3) age 18–65 years; (4) time since brain injury of more than 28 days and less than 1 year; (5) stable vital signs and a clinical condition permitting completion of the 30-min acupuncture procedure and paired pre- and post-acupuncture fNIRS recordings; and (6) written informed consent provided by a legally authorized representative.

The exclusion criteria were: (1) uncontrolled epilepsy or recent frequent seizures; (2) scalp lesions, unstable skull defects, or other cranial conditions that precluded reliable placement of the fNIRS optodes; (3) localized infection, ulceration, bleeding, or severe skin irritation at the acupuncture sites or scalp regions used for fNIRS acquisition; (4) clinically significant arrhythmia or unstable autonomic or cardiovascular status; (5) general anesthesia or deep sedation at the time of assessment; (6) an acute critical condition, including acute myocardial infarction, pulmonary embolism, sepsis, severe pulmonary infection, intracranial infection, or brain herniation; and (7) recent initiation or substantial adjustment of sedative medication, or another concurrent intervention expected to acutely alter arousal or cerebral hemodynamics during the pre−/post-acupuncture recording window.

After enrollment, withdrawal or study discontinuation could occur if: (1) the legally authorized representative withdrew consent or requested withdrawal; (2) marked clinical deterioration or a serious adverse event occurred; or (3) the investigator determined that continued participation or completion of the acupuncture procedure or either fNIRS recording was unsafe or infeasible. A dropout was defined as an enrolled participant who did not complete the acupuncture procedure or either of the paired fNIRS assessments for reasons other than consent withdrawal or investigator-initiated discontinuation. The paired analysis required complete pre- and post-acupuncture fNIRS recordings. All 30 participants in the present cohort completed the acupuncture procedure and both recordings; no withdrawal, study discontinuation, or dropout occurred, and all 30 participants were retained in the final paired analysis.

Because the most closely related acupuncture–fNIRS studies in patients with prolonged DOC evaluated cortical activation and selected regional functional connections but did not provide a directly transferable paired effect-size estimate for the graph-theoretical outcomes examined here (Liu et al., 2022; Xin et al., 2022), no formal *a priori* sample-size calculation based on an assumed expected effect was performed. For the available analytical sample of 30 participants, an effect-size sensitivity analysis was conducted using G*Power 3.1 (Faul et al., 2007). For a two-sided paired-samples *t*-test with α = 0.05 and 80% power, the minimum detectable standardized mean difference in paired change scores was Cohen’s *d_z_* = 0.53. This sensitivity estimate applies to an individual paired comparison before correction for multiple testing and should not be interpreted as the power of all FDR-adjusted analyses.

Acupuncture was administered as an adjunctive intervention rather than as the sole clinical treatment. Participants continued to receive routine medical treatment, nursing care, rehabilitation, and nutritional support according to individual clinical needs. The study protocol did not mandate the initiation, withdrawal, or adjustment of clinically indicated care for the experimental session. The paired fNIRS recordings were obtained within a single session comprising a 10-min pre-acupuncture resting-state recording, the 30-min acupuncture procedure, and a 10-min post-acupuncture resting-state recording immediately afterward. Participants were assessed using the same fNIRS setup and, whenever clinically feasible, under comparable positioning, environmental, and clinical conditions. Routine and clinically necessary bedside care was not withheld; the occurrence and timing of such procedures were not prospectively standardized or analyzed as experimental variables.

### Acupuncture intervention

Acupuncture was performed at Shuigou (GV26), Yintang (EX-HN3), and Baihui (GV20), with one needle inserted at each acupoint (three needles per participant). The locations of GV20 and GV26 were determined according to the Chinese National Standard GB/T 12346–2021, Nomenclature and Location of Meridian Points, whereas EX-HN3 was located according to GB/T 40997–2021, Nomenclature and Location of Extra Points in Common Use. Basic filiform-needle insertion and manual manipulation were described with reference to GB/T 21709.20–2009 and GB/T 21709.21–2013, respectively, and the intervention was reported in accordance with the STRICTA recommendations (MacPherson et al., 2010; Standardization Administration of China, 2009, 2013, 2021a, 2021b). GV26 was located at the junction of the upper one-third and lower two-thirds of the philtrum; EX-HN3 at the midpoint between the medial ends of the eyebrows; and GV20 on the anterior median line, 5 proportional bone cun (B-cun) superior to the anterior hairline.

After routine skin disinfection, sterile single-use filiform acupuncture needles (Hwato, Suzhou Medical Supplies Factory Co., Ltd., Suzhou, China; 0.25 × 40 mm) were inserted manually. The needles were supplied as routine sterile clinical consumables; individual lot numbers were not prospectively recorded in the study database and were therefore unavailable for retrospective reporting. Point-specific insertion parameters were specified with reference to published standardized protocols using the same cranial acupoints (Bai et al., 2024; Lin et al., 2025). GV20 was inserted subcutaneously from anterior to posterior at approximately 15° to the scalp surface to a depth of 0.5–0.8 cun (approximately 12.5–20 mm). GV26 was inserted obliquely upward toward the nasal septum at approximately 30°–45° to a depth of 10–15 mm. EX-HN3 was inserted obliquely downward toward the nasal root at approximately 15°–30° to a depth of 10–15 mm.

An even reinforcing-reducing technique (ping bu ping xie) was applied using gentle, uniform lifting-thrusting and bidirectional twirling with approximately equal amplitude and force, without preferential reinforcing or reducing stimulation. Because patients with DOC could not reliably report subjective needle sensations, patient-reported deqi was not required or assessed; manipulation intensity was adjusted according to local tissue response, clinical observation, and safety considerations. No electrical stimulation was used. Needles were retained for 30 min and removed before the post-acupuncture fNIRS recording. Each participant received one acupuncture session administered by qualified acupuncturists with clinical experience in neurological rehabilitation. Vital signs were monitored throughout the procedure, and adverse events were recorded.

### fNIRS acquisition

Resting-state fNIRS signals were acquired before and after acupuncture. For each participant, oxygenated hemoglobin (HbO) time series were obtained from 10 cortical regions of interest: left dorsolateral prefrontal cortex (left-DLPFC), right dorsolateral prefrontal cortex (right-DLPFC), left Broca area, right Broca area, left frontal cortex, right frontal cortex, left primary somatosensory cortex (left-PSC), right primary somatosensory cortex (right-PSC), left premotor cortex (left-PMC), and right premotor cortex (right-PMC). Each recording lasted 10 min at a sampling rate of 20 Hz, yielding 12,000 time points per condition. HbO was selected as the primary hemodynamic signal because it is generally more sensitive than deoxygenated hemoglobin to task- and state-related cortical changes in fNIRS recordings (Pinti et al., 2020; Pinti et al., 2019).

The selection of the 10 cortical regions of interest was hypothesis-driven and constrained by the spatial coverage of the bedside fNIRS montage. Bilateral dorsolateral prefrontal, frontal, Broca, primary somatosensory, and premotor regions were prioritized because they support executive and integrative processing, language-related processing, somatosensory integration, motor preparation, and behavioral responsiveness relevant to residual cortical function in DOC. The parietal lobe was not entirely excluded: the bilateral primary somatosensory regions are located in the postcentral gyrus of the parietal lobe. However, the available montage did not cover posterior parietal association regions, including the precuneus and inferior parietal lobule, or the temporal cortex. Accordingly, the network analyses characterize the recorded cortical system rather than the whole cerebral cortex.

### Preprocessing of fNIRS signals

All analyses were performed on HbO signals. For each participant, resting-state HbO data were obtained from 10 cortical regions before acupuncture and again after acupuncture. Each recording lasted 10 min with a sampling rate of 20 Hz, resulting in 12,000 time points per condition. Prior to quantitative analysis, each subject- and region-specific time series was linearly detrended to reduce slow baseline drift. No participant was excluded from the pre−/post-paired fNIRS analysis.

### Spectral analysis and regional fluctuation measures

To characterize spontaneous low-frequency hemodynamic fluctuations, power spectral density was estimated for each detrended HbO time series using Welch’s method. Based on the conventional frequency range of resting-state fNIRS oscillations, the low-frequency band of interest was defined as 0.01–0.06 Hz. Regional low-frequency fluctuation measures were then derived for each channel, including the standard deviation of the time series, amplitude of low-frequency fluctuation (ALFF), and fractional ALFF (fALFF). These indices were used to quantify pre−/post-acupuncture differences in the amplitude characteristics of spontaneous hemodynamic activity at the regional level.

### Functional connectivity analysis

Resting-state functional connectivity was calculated separately for the pre-acupuncture and post-acupuncture conditions. For each participant, pairwise Pearson correlation coefficients were computed between all pairs of the 10 HbO regional time series, generating a 10 × 10 connectivity matrix for each condition. Correlation coefficients were then transformed to Fisher’s *z* values for statistical comparison across conditions. Group-level connectivity patterns were visualized using mean connectivity matrices and post-minus-pre difference matrices.

In addition to edge-wise analysis, a mean positive functional connectivity metric was computed for each participant by averaging all positive functional connections within the whole-brain matrix. This measure was used as a global summary index of overall network coupling strength.

### Graph-theoretical network analysis

To further characterize network-level differences between the pre- and post-acupuncture recordings, graph-theoretical measures were derived from positive-weighted functional connectivity matrices. Negative connections were excluded from graph construction so that the resulting network metrics specifically reflected positively coordinated cortical interactions. For each participant and condition, the following global metrics were calculated: global efficiency, clustering coefficient, and positive-edge density. Global efficiency was used to quantify the capacity for large-scale information integration across the network, whereas the clustering coefficient reflected the degree of local interconnectedness or segregation. Positive-edge density was defined as the proportion of region pairs showing positive connectivity values.

At the nodal level, nodal strength was calculated for each of the 10 regions as the sum of positive connection weights linked to that node. This metric was used to identify cortical regions showing the largest post-acupuncture changes in network centrality.

### Subnetwork analysis

To examine whether pre−/post-acupuncture differences varied across specific large-scale subnetworks, several predefined subnetwork connectivity metrics were computed by averaging Fisher *z*-transformed functional connectivity values within or between anatomically grouped regions. The anterior network comprised bilateral DLPFC, Broca, and frontal regions, whereas the posterior network comprised bilateral PSC and PMC regions. Based on this anatomical grouping, the following subnetwork measures were calculated: posterior connectivity, within-left-hemisphere connectivity, within-right-hemisphere connectivity, interhemispheric connectivity, and fronto-posterior connectivity. Posterior connectivity represented the average connectivity among posterior regions; within-left- and within-right-hemisphere connectivity represented the average connectivity among regions within the same hemisphere; interhemispheric connectivity represented average connectivity between left- and right-hemispheric regions; and fronto-posterior connectivity represented average connectivity between anterior and posterior regions.

### Clinical stratification and exploratory analyses

Clinical information was available for 29 of the 30 participants and included age, sex, baseline state, time since brain injury, and baseline clinical score. Baseline state was categorized as VS/UWS or MCS. These clinical variables were incorporated into exploratory analyses to assess potential sources of interindividual heterogeneity in post-acupuncture network changes.

The repeated CRS-R assessments described above were used to establish diagnostic status before enrollment. The protocol did not include an immediate post-acupuncture CRS-R assessment or longitudinal CRS-R follow-up as study outcomes; accordingly, behavioral scale scores were not analyzed as paired efficacy endpoints.

First, subgroup analyses were performed by comparing changes in key network metrics between VS/UWS and MCS patients and between male and female patients. Second, Spearman correlation analyses were used to evaluate associations between continuous clinical variables and both baseline network measures and post-acupuncture change scores. Third, multivariable linear regression models were constructed to explore whether age, sex, baseline state, time since brain injury, and baseline clinical score independently predicted changes in mean functional connectivity, global efficiency, and clustering coefficient. Because these analyses were exploratory and the sample size was limited, they were interpreted cautiously and used primarily to characterize heterogeneity in post-acupuncture change scores rather than to establish definitive clinical predictors.

### Statistical analysis

All statistical analyses were performed at the subject level using paired pre−/post-acupuncture comparisons. For whole-network metrics, nodal strength, regional fluctuation measures, subnetwork measures, and edge-wise connectivity values, pre- and post-acupuncture values were compared using paired *t* tests. Effect sizes were quantified as Cohen’s *d*z for paired data.

To control for multiple comparisons, false discovery rate (FDR) correction was applied separately within each family of tests, including global network metrics, nodal metrics, regional fluctuation measures, subnetwork metrics, and edge-wise connectivity analyses. Statistical significance was defined as a two-tailed *p* < 0.05 before correction, with FDR-corrected *q* < 0.05 considered significant after multiple-comparison adjustment.

For the exploratory clinical analyses, between-group comparisons of change scores were performed using independent-samples tests as appropriate, and associations between clinical variables and network measures were assessed using Spearman rank correlation. Multivariable regression results were summarized using standardized regression coefficients and 95% confidence intervals. Because these clinical analyses were secondary and exploratory, non-significant findings were not overinterpreted.

The participant flow and single-session experimental protocol are summarized in Figure 1, and the fNIRS acquisition, preprocessing, and analysis pipeline is summarized in Figure 2.

**Figure 1:**
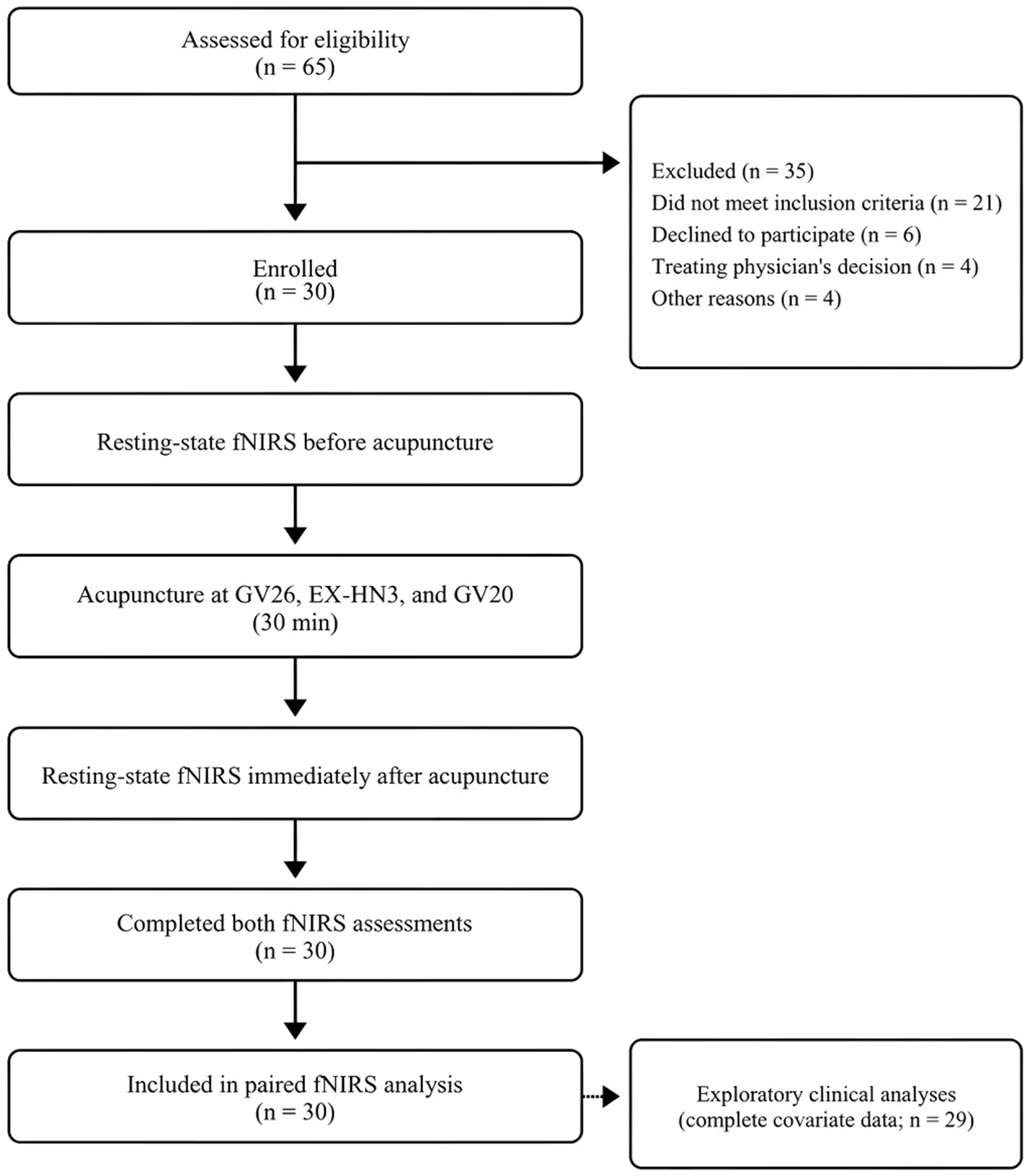
Participant flow through the study. Of 65 patients assessed for eligibility, 35 were excluded and 30 were enrolled. All enrolled participants completed the acupuncture procedure and both resting-state fNIRS recordings and were included in the paired imaging analysis. Clinical covariate data were available for 29 participants, who were included in the exploratory clinical analyses.

**Figure 2:**
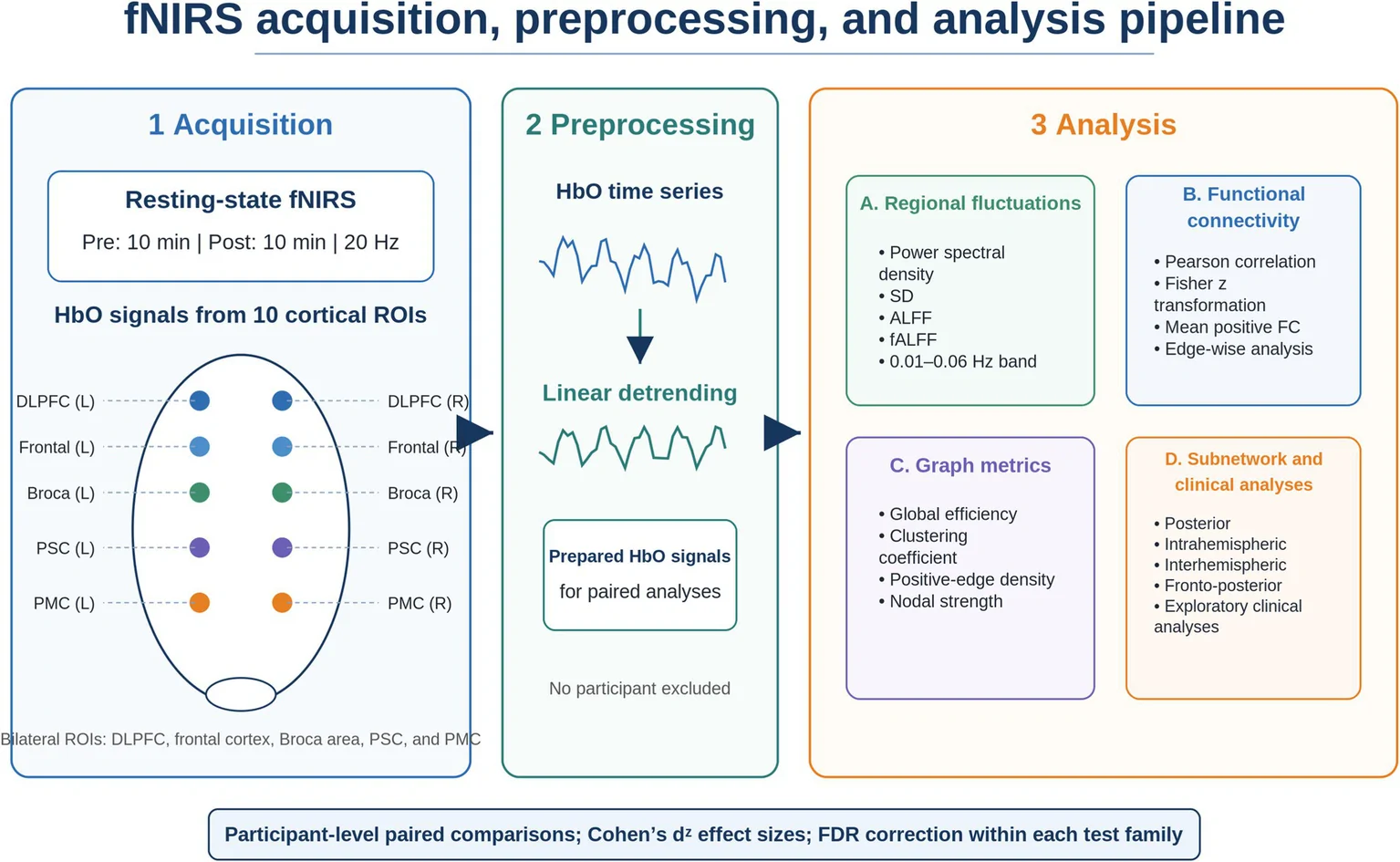
fNIRS acquisition, preprocessing, and analysis pipeline. HbO signals from 10 cortical regions were linearly detrended and analyzed using regional fluctuation, functional connectivity, graph-theoretical, subnetwork, and exploratory clinical analyses.

## Results

### Participant characteristics

Thirty patients with disorders of consciousness were included in the paired pre−/post-acupuncture resting-state fNIRS analysis. The HbO dataset comprised 10 min of resting-state recording before acupuncture and 10 min after acupuncture for each participant (20 Hz; 12,000 time points per condition; 10 cortical regions). Clinical information was available for 29 participants. The mean age was 49.5 ± 13.3 years, 23 were male and 7 were female, 13 were classified as VS/UWS and 17 as MCS, the mean time since brain injury was 7.2 ± 3.1 months, and the baseline Coma Recovery Scale–Revised (CRS-R) total score was 7.8 ± 2.2 (Table 1).

**Table 1:** Demographic and clinical characteristics of the 30 participants.

Characteristic	Value
Age, years, mean ± SD	49.5 ± 13.3
Sex, n (%)	Male 23 (76.7); female 7 (23.3)
Diagnosis, *n*	VS/UWS 13; MCS 17
Etiology, *n*	traumatic brain injury 8; cerebral hemorrhage 9; anoxic–ischemic encephalopathy 13
Time since brain injury, months, mean ± SD	7.2 ± 3.1
Baseline CRS-R total score, mean ± SD	7.8 ± 2.2

Clinical information was available for 29 participants. CRS-R, Coma Recovery Scale–Revised; VS/UWS, vegetative state/unresponsive wakefulness syndrome; MCS, minimally conscious state.

### Group-level functional connectivity was broadly higher after acupuncture

To visualize overall changes in resting-state cortical coupling, group-averaged functional connectivity matrices were constructed for the pre-acupuncture and post-acupuncture conditions (Figures 3A,B). Compared with the pre-acupuncture condition, the post-acupuncture matrix showed a broadly increased pattern of positive connectivity across multiple cortical regions. The post-minus-pre difference matrix further demonstrated that the post-acupuncture increase was spatially distributed rather than confined to a single pair of regions (Figure 3C). The most prominent increases were observed in connections involving frontal and posterior regions, including fronto-posterior and cross-regional interactions. These matrix-level observations indicate a broad post-acupuncture increase in intrinsic cortical coordination across the recorded regions.

**Figure 3:**
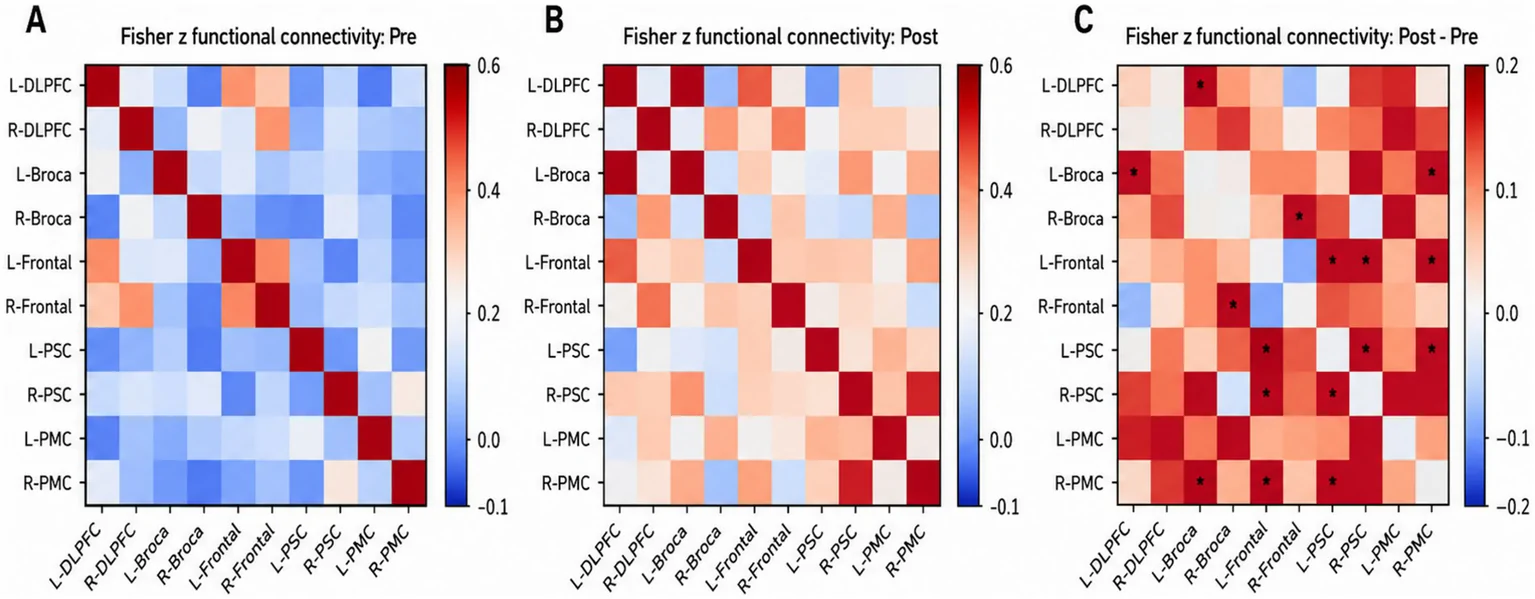
Group-level functional connectivity matrices before and after acupuncture. **(A)** Mean resting-state functional connectivity matrix in the pre-acupuncture condition. **(B)** Mean resting-state functional connectivity matrix in the post-acupuncture condition. **(C)** Post-minus-pre connectivity difference matrix. Warmer colors indicate higher Fisher z-transformed connectivity values in the post-acupuncture condition. The overall pattern shows widespread post-minus-pre increases, especially in connections involving frontal and posterior regions.

### Large-scale resting-state functional integration was higher after acupuncture

To characterize resting-state cortical reorganization after acupuncture, we quantified low-frequency HbO functional connectivity in the 0.01–0.06 Hz band and derived graph-theoretical metrics from positive-weighted connectivity matrices. At the whole-network level, acupuncture significantly increased mean positive functional connectivity (pre: 0.231, post: 0.311, paired *p* = 0.021, FDR-corrected *q* = 0.041, Cohen’s *d*z = 0.447), global efficiency (pre: 0.287, post: 0.364, *p* = 0.016, *q* = 0.041, *d*z = 0.468), and clustering coefficient (pre: 0.338, post: 0.428, *p* = 0.031, *q* = 0.041, *d*z = 0.422) (Figure 4). In contrast, the density of positive edges did not show a significant change, suggesting that the post-acupuncture increase was mainly driven by strengthened coupling rather than a simple increase in the number of detectable positive connections.

**Figure 4:**
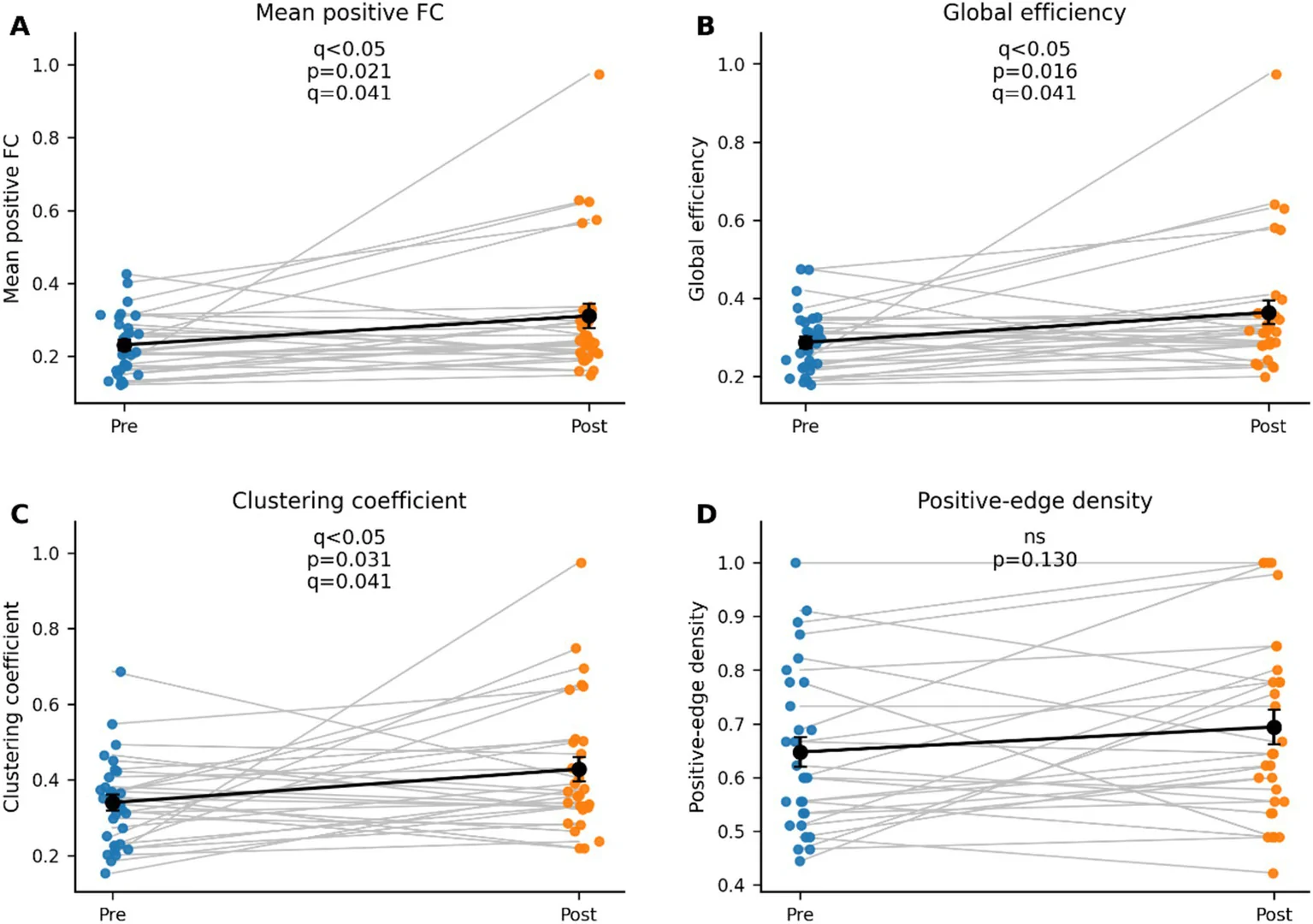
Global network measures before and after acupuncture. **(A)** Mean positive functional connectivity. **(B)** Global efficiency. **(C)** Clustering coefficient. **(D)** Positive-edge density. Gray lines connect the paired pre- and post-acupuncture measurements for each participant, and black symbols and lines represent the group means ± SEM. Following FDR correction, mean positive functional connectivity, global efficiency, and clustering coefficient were significantly higher after acupuncture, whereas positive-edge density did not differ significantly.

### Post-acupuncture changes were most prominent in posterior, left intrahemispheric, and fronto-posterior subnetworks

Subnetwork-level analyses revealed a nonuniform pattern of connectivity changes following acupuncture (Figure 5). Among the predefined subnetworks, within-left-hemisphere connectivity showed a significant increase after acupuncture at the uncorrected level (*p* = 0.038). Posterior connectivity also increased significantly (*p* = 0.011), as did fronto-posterior connectivity (*p* = 0.043). In contrast, within-right-hemisphere (*p* = 0.140), interhemispheric (*p* = 0.061), homologous (*p* = 0.527), anterior (*p* = 0.235), and global connectivity (*p* = 0.058) did not reach nominal statistical significance.

However, none of these subnetwork-level effects remained significant after Benjamini-Hochberg false discovery rate correction across the eight predefined connectivity measures (all FDR-adjusted *q* > 0.05). Therefore, the increases in posterior, left intrahemispheric, and fronto-posterior connectivity should be interpreted as nominal or trend-level effects rather than statistically robust findings after correction for multiple comparisons. Overall, these results suggest a tendency toward enhanced posterior and anterior-posterior cortical coordination after acupuncture, but this spatial pattern requires confirmation in larger cohorts.

**Figure 5:**
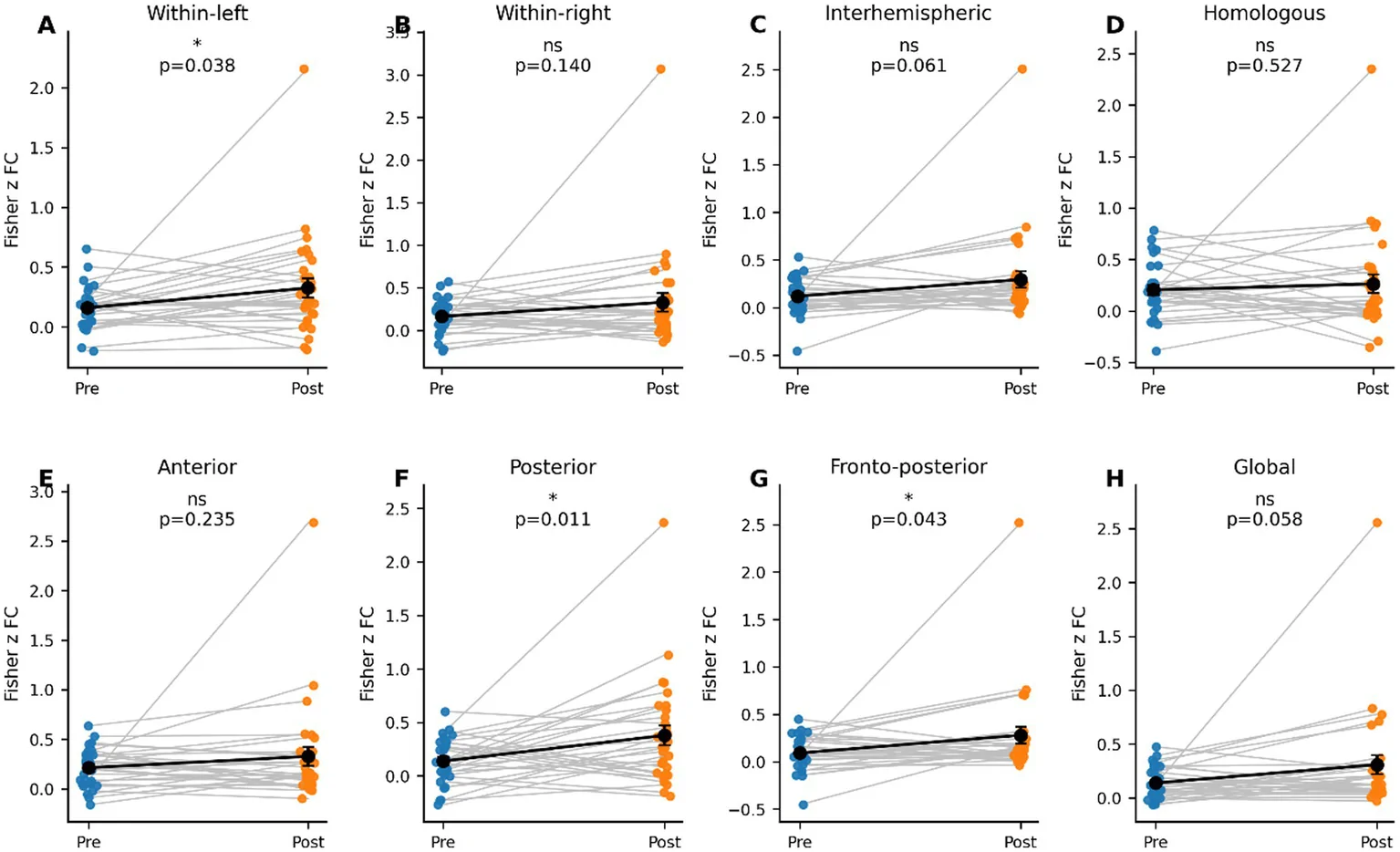
Subnetwork functional connectivity changes following acupuncture. **(A)** Within-left-hemisphere connectivity. **(B)** Within-right-hemisphere connectivity. **(C)** Interhemispheric connectivity. **(D)** Homologous connectivity. **(E)** Anterior connectivity. **(F)** Posterior connectivity. **(G)** Fronto-posterior connectivity. **(H)** Global connectivity. Gray lines connect the paired measurements for each participant, and black symbols and lines represent the group means ± SEM. Values are Fisher z-transformed Pearson correlation coefficients. Asterisks indicate nominal significance before correction (*p* < 0.05), whereas “ns” indicates *p* ≥ 0.05. None of the eight subnetwork comparisons remained significant after Benjamini–Hochberg FDR correction.

### Post-acupuncture nodal-strength increases were centered on language-related and sensorimotor-associated regions

At the nodal level, several regions showed robust increases in nodal strength after acupuncture (Figure 6). The strongest effect was observed in the left Broca area (pre: 1.989, post: 3.018, *p* = 0.002, *q* = 0.023, *d*z = 0.609). Significant increases were also detected in the left PSC (pre: 1.858, post: 2.764, *p* = 0.007, *q* = 0.035, *d*z = 0.528), right PSC (pre: 2.105, post: 3.054, *p* = 0.011, *q* = 0.035, *d*z = 0.497), and right PMC (pre: 1.850, post: 2.746, *p* = 0.019, *q* = 0.047, *d*z = 0.453). Additional increases were observed in the left PMC, left frontal, and bilateral DLPFC regions, but these did not survive correction for multiple comparisons. Overall, the nodal pattern suggests that the post-acupuncture reorganization was concentrated in a fronto-language-sensorimotor network.

**Figure 6:**
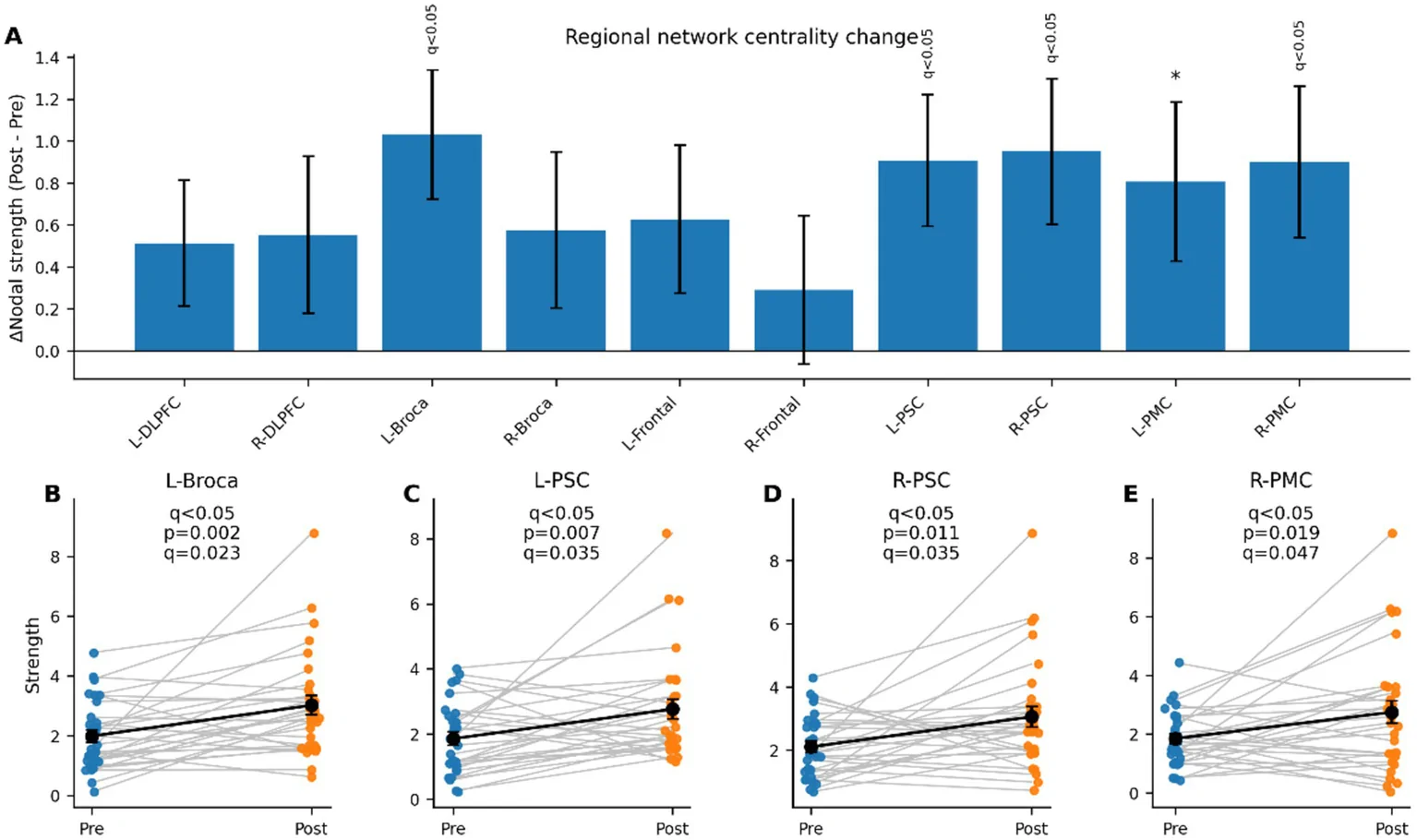
Nodal strength changes across cortical regions. **(A)** Mean post-minus-pre change in nodal strength across the 10 fNIRS regions. **(B)** Paired nodal strength values for the left Broca area. **(C)** Paired nodal strength values for the left primary somatosensory cortex (PSC). **(D)** Paired nodal strength values for the right PSC. **(E)** Paired nodal strength values for the right premotor cortex (PMC). Significant post-acupuncture increases were observed in panels B–E after FDR correction.

### Edge-wise connectivity changes showed a distributed but not individually FDR-significant pattern

At the edge level, several connections exhibited nominally higher post-acupuncture connectivity, including left DLPFC–left Broca, left frontal–right PSC, left frontal–right PMC, right Broca–right frontal, and left PSC–right PMC. However, none of the individual edges survived FDR correction across all tested pairs. This pattern indicates that the pre−/post-acupuncture difference was more apparent at the systems level, through coherent shifts in global and regional network organization, than through isolated pairwise connections.

### Regional low-frequency HbO fluctuations showed trends without robust FDR-corrected differences

Signal-variability and amplitude analyses revealed a general tendency toward increased low-frequency spontaneous fluctuation after acupuncture, particularly in the left Broca region and right PMC. For example, ALFF in the left Broca area increased numerically from 0.000035 to 0.000220 (*p* = 0.0154), and temporal standard deviation also increased in the same region (*p* = 0.0149). Nevertheless, these regional fluctuation measures did not survive FDR correction (Figure 7), indicating that the most reliable pre−/post-acupuncture differences in the current dataset were observed in functional coordination rather than local amplitude alone.

**Figure 7:**
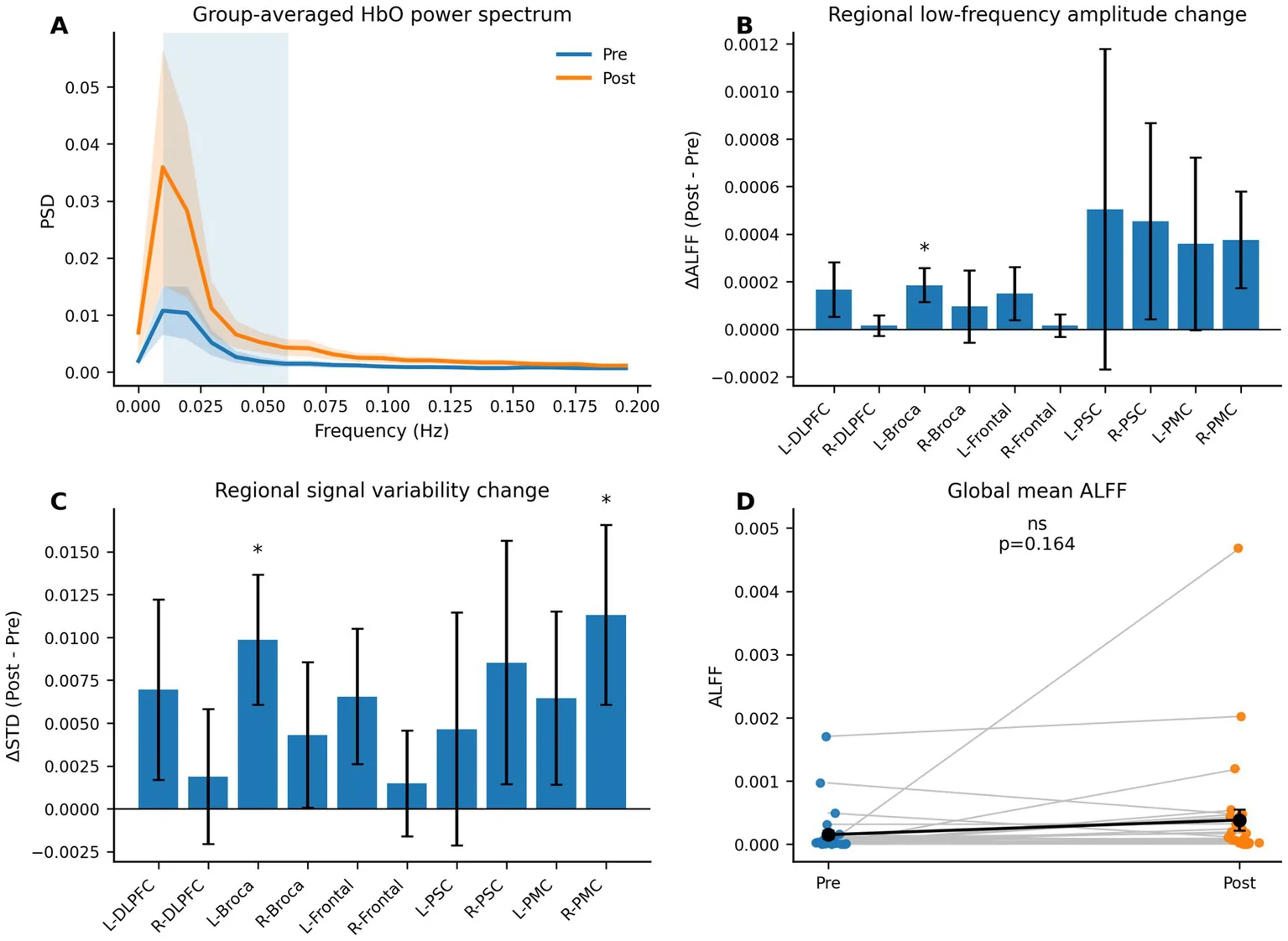
Resting-state HbO signal characteristics before and after acupuncture. **(A)** Group-averaged HbO power spectra under the pre- and post-acupuncture conditions. **(B)** Post-minus-pre changes in regional amplitude of low-frequency fluctuations (ΔALFF) across the 10 fNIRS regions. **(C)** Post-minus-pre changes in regional signal variability, quantified as the temporal standard deviation (ΔSTD). **(D)** Paired pre- and post-acupuncture global mean ALFF values. Asterisks indicate nominal significance before correction (*p* < 0.05), whereas “ns” indicates a nonsignificant difference. None of the regional ALFF or signal-variability comparisons remained significant after FDR correction.

### Exploratory clinical analyses showed limited evidence for stable predictors of post-acupuncture network change

To further examine interindividual heterogeneity, we incorporated age, sex, baseline state, time since brain injury, and baseline clinical score into exploratory analyses. The overall post-acupuncture increases in mean functional connectivity, global efficiency, and clustering remained evident after adding clinical information. When stratified by baseline state, both the VS/UWS and MCS groups showed post-acupuncture network increases, with numerically larger increases in the MCS group for mean functional connectivity, global efficiency, and clustering, but none of these between-group differences reached significance (all *p* > 0.35). Similarly, no significant sex-related differences were found, although clustering tended to increase more in males than in females (*p* = 0.071).

Spearman analyses did not detect significant associations between age, time since brain injury, or baseline score and the main post-acupuncture network changes after FDR correction. A trend-level negative association was observed between time since brain injury and the increase in within-left-hemisphere connectivity (rho = −0.372, *p* = 0.0468), but this did not survive correction. Multivariable linear models likewise showed limited explanatory power and did not identify any stable independent predictors of changes in mean functional connectivity, global efficiency, or clustering. Collectively, these exploratory analyses suggest that the observed post-acupuncture resting-state network increases may be relatively general across the current cohort, whereas the sources of individual variability remain to be clarified in larger samples.

## Discussion

In this within-subject resting-state fNIRS study, we characterized immediate cortical network differences surrounding a single acupuncture session in patients with DOC. The principal finding was a coordinated shift across the recorded cortical system rather than an isolated change in one region. Mean positive functional connectivity, global efficiency, and clustering coefficient were higher after the acupuncture session, whereas positive-edge density was unchanged. Thus, the observed pattern is best regarded as an acute reconfiguration of the recorded residual cortical network, rather than as evidence of acupuncture-specific therapeutic efficacy or a simple focal activation effect.

The two most closely related fNIRS studies provide important context for these findings. Xin et al. (2022) monitored 16 patients with DOC during acupuncture at GV26 and reported increased prefrontal HbO together with stronger left-sided cortical connectivity, whereas Liu et al. (2022) randomized 28 patients with prolonged DOC to PC6 acupuncture or sham acupuncture and focused on activation and selected resting-state connections among the DLPFC, primary motor cortex, and primary somatosensory cortex. The present study differs in its three-point craniofacial protocol, immediate pre−/post-session resting-state design, 10-region montage, and use of graph-theoretical measures. These differences make the studies complementary rather than directly interchangeable. The sham-controlled design of Liu et al. provides stronger evidence regarding stimulation specificity, whereas the present analysis more explicitly describes distributed network topology within the recorded cortical system. Taken together, the available fNIRS evidence suggests that acupuncture-associated brain responses in DOC may be expressed at both regional and large-scale network levels.

This network-level interpretation is also consistent with a broader methodological shift in acupuncture neuroscience from average regional activation toward distributed and state-sensitive neural representations. Recent EEG studies have quantified acupuncture-related spectral power and functional connectivity, separated periodic from aperiodic activity, decoded needle-twirling and retention states using transformer models, and represented acupuncture manipulations or somatic perception in low-dimensional neural manifolds and spatiospectral latent spaces (Yu et al., 2018; Yu et al., 2024; Yu et al., 2025; Rao et al., 2025; Yu et al., 2026). These studies involved different populations, stimulation protocols, and electrophysiological signals and therefore do not validate the present fNIRS findings directly. Nevertheless, they support examining acupuncture responses as distributed patterns rather than single-region effects. The current results extend this conceptual perspective to bedside hemodynamic network organization in DOC: the post-session differences were more coherent in global and subnetwork measures than in isolated edges or local low-frequency amplitude indices.

At the whole-network level, the coexistence of higher global efficiency and higher clustering coefficient, without a significant change in positive-edge density, suggests stronger weighting of existing positive connections rather than a nonspecific increase in the number of positive connections. This pattern is relevant to contemporary accounts in which consciousness depends on a dynamic balance between distributed integration and locally organized processing (Jang et al., 2024). Spatially, the largest differences involved posterior, within-left-hemisphere, and fronto-posterior connectivity, with nodal-strength increases in the left Broca area, bilateral primary somatosensory cortices, and right premotor cortex. These regions and pathways participate in language-related processing, somatosensory integration, motor preparation, and frontoposterior communication, which are frequently disrupted in DOC (Li et al., 2023; Panda et al., 2022; Ihalainen et al., 2024; Bodien et al., 2024; Amiri et al., 2023; Xu et al., 2024). The convergence of global, subnetwork, and nodal findings is therefore compatible with recruitment of a distributed fronto-language–sensorimotor system; however, it does not demonstrate recovery of consciousness or identify the direction of information flow.

The selection of GV20, GV26, and EX-HN3 reflects their use in wake-promoting acupuncture approaches for neurological dysfunction and DOC protocols (Xin et al., 2022; Lin et al., 2025). In traditional acupuncture theory, craniofacial and midline Governor Vessel points are commonly incorporated into approaches described as “awakening the mind” or “opening the orifices.” This traditional rationale should not be interpreted as direct evidence for a modern neural mechanism. From a contemporary neurobiological perspective, needling the scalp and face provides somatosensory input through cranial and upper cervical afferent pathways, which may influence cortical activity through ascending sensory, brainstem, thalamocortical, and autonomic interactions. The thalamus and brainstem are central to arousal regulation in DOC (Shine et al., 2023; Huang et al., 2024; He et al., 2025), and the observed involvement of somatosensory, premotor, and fronto-posterior networks is compatible with distributed sensory-network engagement. However, because fNIRS measures superficial cortical hemodynamics, the present study cannot directly test these proposed subcortical routes.

The present design also cannot establish acupoint specificity. All three acupoints were administered as one combined intervention, and no sham-acupuncture, non-acupoint, alternative-acupoint, or separate single-point condition was included. Consequently, the observed differences cannot be attributed to GV20, GV26, or EX-HN3 individually, nor can they be distinguished from broader somatosensory stimulation, spontaneous hemodynamic fluctuation, repeated-measurement or order effects, adaptation to the recording environment, systemic physiological variation, or concomitant bedside care. Acupuncture was provided in addition to ongoing routine treatment, and clinically necessary care was not withheld. Future studies should therefore incorporate time-matched sham or non-acupoint controls and, where feasible, separate acupoint arms to distinguish general needling effects from point-combination and point-specific effects.

Behavioral efficacy and durability were not evaluated in the present protocol. Repeated CRS-R assessments were used to establish diagnostic status before enrollment, but neither an immediate post-session CRS-R assessment nor longitudinal behavioral follow-up was prespecified as an outcome. A single fNIRS recording obtained immediately after one session cannot determine whether the network differences were brief, persisted across time, or represented the beginning of durable neuroplastic reorganization. We therefore interpret the findings as acute cortical network responsivity within the recorded system. Such responsivity may be compatible with residual network recruitability, but this remains a hypothesis rather than evidence of lasting plasticity or clinical improvement. Controlled longitudinal studies should combine serial fNIRS with repeated CRS-R assessments before, during, and after a multi-session treatment course and at follow-up to determine whether early network differences persist, correlate with concurrent behavioral change, or predict later recovery.

Other limitations concern spatial coverage, physiological control, and generalizability. Although the montage sampled bilateral primary somatosensory cortex within the parietal lobe, it did not cover posterior parietal association regions, such as the precuneus and inferior parietal lobule, or the temporal cortex. fNIRS also cannot directly assess the thalamus, brainstem, or other deep structures. In addition, systemic physiological signals and the timing of routine bedside procedures were not prospectively modeled as experimental variables. The sample was modest and etiologically and diagnostically heterogeneous, limiting power to identify stable response predictors. The montage did not include short-separation channels; restricting the analysis to the 0.01–0.06 Hz band attenuates cardiac and respiratory contributions, but extracerebral scalp hemodynamics and low-frequency systemic oscillations cannot be fully excluded as contributors to the observed connectivity differences. In addition, the standardized effects for the significant global metrics (Cohen’s *dz* = 0.42–0.47) were smaller than the minimum detectable effect estimated for an individual paired comparison (*dz* = 0.53); these estimates should therefore be regarded as preliminary and require confirmation in larger cohorts. Future multicenter studies should use high-density or whole-head fNIRS, record systemic physiology and concomitant care prospectively, combine fNIRS with EEG and structural or functional MRI, and predefine analyses stratified by diagnosis and etiology. Such designs would provide a more complete assessment of cortical–subcortical mechanisms, reproducibility, and clinical relevance.

In summary, the present findings extend prior regional fNIRS observations and recent neural-representation approaches by describing immediate systems-level differences in the recorded cortical network after a single acupuncture session. The most consistent changes involved higher global integration, stronger posterior and fronto-posterior connectivity, and higher nodal strength in language-related and sensorimotor-associated regions. These results support bedside fNIRS as a feasible method for characterizing acute network responsivity in DOC, while the absence of matched controls, behavioral efficacy outcomes, longitudinal follow-up, and whole-brain coverage requires a cautious interpretation.

## Conclusion

A single acupuncture session at Shuigou, Yintang, and Baihui was followed by significantly higher resting-state cortical functional connectivity and graph-theoretical network integration in patients with DOC. The largest pre−/post-acupuncture differences were observed at the global, subnetwork, and nodal levels, particularly in posterior, left intrahemispheric, fronto-posterior, language-related, and sensorimotor-associated regions. These results characterize an immediate systems-level cortical network pattern temporally associated with acupuncture, rather than establishing acupuncture-specific neuromodulation or clinical benefit. Controlled longitudinal studies are required to determine specificity, clinical significance, and prognostic value.

## Data Availability

The raw data supporting the conclusions of this article will be made available by the authors, without undue reservation.
